# Comparative Study on Interdental Papillae Regeneration: Leukocyte Platelet-Rich Fibrin By-product versus Hyaluronic Acid Injections in Modified Open Gingival Embrasure Model

**DOI:** 10.1055/s-0045-1802948

**Published:** 2025-05-01

**Authors:** Rezmelia Sari, Usi Sukorini, Heni Susilowati, Suryono Suryono

**Affiliations:** 1 Doctor of Dental Medicine Study Program, Faculty of Dentistry, Universitas Gadjah Mada, Yogyakarta, Indonesia; 2 Department of Periodontology, Faculty of Dentistry, Universitas Gadjah Mada, Yogyakarta, Indonesia; 3 Department of Clinical Pathology and Laboratory Medicine, Faculty of Medicine, Public Health, and Nursing, Universitas Gadjah Mada, Yogyakarta, Indonesia; 4 Department of Oral Biology, Faculty of Dentistry, Universitas Gadjah Mada, Yogyakarta, Indonesia

**Keywords:** cell proliferation, gingiva, hyaluronic acid, open gingival embrasure, platelet-rich fibrin, regeneration

## Abstract

**Objectives:**

This study aims to compare the regenerative effects of various by-products of human leukocyte platelet-rich fibrin (L-PRF), including L-PRF exudate, concentrated PRF (C-PRF), and a mixture of the two, with hyaluronic acid (HA) specifically for interdental papillae reconstruction.

**Materials and Methods:**

The L-PRF was obtained by centrifuging 10 mL of human blood in a fixed-angle centrifuge at 2,700 rpm for 12 minutes. After centrifugation, the L-PRF layer was separated, and platelet and leukocyte counts were performed. An *in vivo* study was conducted using Sprague-Dawley rats subjected to a modified open gingival embrasure (OGE) model for 7 days. Once the OGE was established, 20 µL of L-PRF exudate ( *n* = 3), C-PRF ( *n* = 3), a combination of L-PRF exudate and C-PRF ( *n* = 3), HA ( *n* = 3), and phosphate-buffered saline ( *n* = 3) were injected 2 mm from the tip of the papillae using a 30G syringe. Clinical parameters, including OGE width and spring papilla distance (SPD), were observed on days 7 and 14. On day 14, histological observations included fibroblast count, blood vessel presence, epithelial width, and collagen density, while proliferating cell nuclear antigen expression was assessed immunohistochemically.

**Statistical Analysis:**

The SPD on day 7, along with all histological and immunohistochemical data, were normally distributed and analyzed by one-way analysis of variance followed by Tukey's honestly significant difference test. In contrast, the Kruskal–Wallis' test was used to analyze the OGE width and SPD on day 14, which was not normally distributed.

**Results:**

The cell counts indicated that most platelets and leukocytes were in the C-PRF layer. The L-PRF membrane by-product increased fibroblast proliferation more effectively than HA ( *p* < 0.05). Only C-PRF significantly enhanced the vascularization and epithelialization of the papillae ( *p* < 0.05). However, the observed cellular and molecular changes increased at day 7 postinjection and did not impact collagen density or interdental papilla height.

**Conclusion:**

The regenerative effect of C-PRF injection is superior to that of HA and other L-PRF by-products, as it promotes papillae regeneration by enhancing fibroblast activity, vascularization, and epithelialization. These findings show the potential impact of L-PRF by-products as a nonsurgical papillae reconstruction treatment.

## Introduction

Inadequate interdental papillae are a challenge in modern dentistry. This condition impacts the function and esthetics of natural teeth, restorations, and implants. Losing interdental papillae increases the risk of periodontal disease and implant failure, as the resulting gaps can accumulate plaque and food debris. Clinically, interdental papillae loss can occur singly or in multiples with various etiologic factors. The prevalence of interdental papillae loss in adults is 38%, based on a study of 119 cases, while in adolescents, the prevalence is 41.9% based on 129 cases. A loss of papillary height of 2 mm poses a significant issue in orthodontics. Both dentists and patients generally agree that a noticeable loss of 3 mm in tooth structure is less esthetically pleasing. Achieving harmony between hard and soft tissues is essential for stabilizing teeth and preventing plaque-related diseases, such as dental caries and periodontal disease. 1

Papillae reconstruction is a complex treatment procedure that requires careful case selection and appropriate techniques and materials. The main goal of papillae reconstruction is to surgically or nonsurgically modify the gingival biotype. The gold standard for rebuilding papillae is surgical intervention combined with a gingival connective tissue graft. However, this method can lead to discomfort for the patient, as it creates a second surgical site at the donor location. 2

In addition to surgical procedures, papillae reconstruction procedures can be performed nonsurgically. A systematic review and meta-analysis showed that hyaluronic acid (HA) injection can be an efficient nonsurgical treatment for narrow interdental papillae loss 3 despite the low success rate of surgical procedures. 4 Nonanimal stabilized HA dermal fillers are currently accessible and have been approved for tissue regeneration by the Food and Drug Administration in more than 60 countries, including Indonesia. However, the clinical effectiveness of HA postinjection treatment results is inconsistent, with side effects such as pain, intermittent edema, erythema, 5 and a burning sensation after the injection. 6 These HA allergic reactions can be treated with injectable platelet-rich fibrin (PRF). 7 However, the quantity of injectable PRF is limited. Further research is required to optimize the use of PRF, particularly for multiple black triangle therapy.

Leukocyte PRF (L-PRF) effectively repairs the loss of interdental papilla. 8 The conventional process for preparing L-PRF is relatively simple. A fixed-angle centrifuge spins human blood at 2,700 rpm for 12 minutes. The subsequent fibrin clot is sliced and compacted to form a dense, robust L-PRF membrane. 9 This membrane is applied using a variety of surgical procedures and has been shown to totally or partially repair the papillae while improving patient comfort. 8

Fibrin clot compression produces by-products, such as L-PRF exudate and buffy coat. Unfortunately, these by-products are often discarded, even though the exudate still contains valuable growth factors, 10 and the buffy coat that was known as concentrated PRF (C-PRF) is more affluent in platelets and leukocytes than injectable PRF, 11 which is essential for regeneration. The effects of injecting the L-PRF by-product layer into interdental papillae have yet to be studied, either *in vivo* or clinically. This study aims to compare the regenerative effects of various types of L-PRF by-products (including L-PRF exudate, C-PRF, and a combination of C-PRF and L-PRF) with HA as a nonsurgical treatment for the reconstruction of interdental papillae. The regeneration parameters were evaluated clinically, histologically, and immunohistochemically.

## Materials and Methods

### PRF Preparation and Cell Count

The research protocol has been approved by the Research Ethics Commission of the Faculty of Dentistry at Gadjah Mada University under the number 8/UN1/KEP/FKG-RSGM/EC/2023. PRF was derived from healthy, nonsmoking individuals with a normal platelet count ranging from 150,000 to 450,000 cells/µL in their blood who were not taking any medications that could influence platelet function.

After signing the consent form, up to 10 mL of venous blood was drawn and placed in a glass tube without anticoagulants. The blood was centrifuged for 12 minutes at 2,700 rpm in a fixed-angle centrifuge. 9 Following centrifugation, the samples were allowed to stand for ∼3 minutes. This study separated the PRF based on the layer produced using scissors and then stored it in a labeled container.

The fibrin clot's middle layer was compressed using a PRF box, resulting in a liquid by-product called L-PRF exudate. The layer above the red blood cell layer is called the buffy coat, or C-PRF, while the layer between the fibrin and the buffy coat comprises a combination of L-PRF and C-PRF. The number of platelets and leukocytes was counted in each layer to ensure that the centrifugation procedure in this study can separate the PRF layer as effectively as in previous research. 11

### *In vivo* Study on Modified Open Gingival Embrasure Model and Data Analysis

**Fig. 1:**
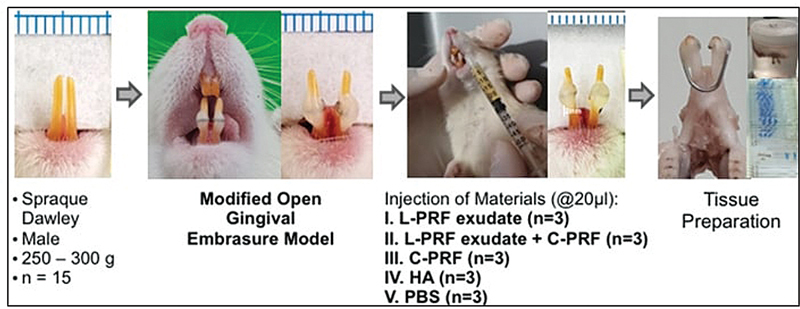
Research flow of *in vivo* study on modified OGE model. The space of mandibular incisors is more expansive after lateral force to confirm OGE. C-PRF, concentrated platelet-rich fibrin; HA, hyaluronic acid; L-PRF, leucocyte platelet-rich fibrin; OGE, open gingival embrasure; PBS, phosphate-buffered saline.

This *in vivo* study employed a modified open gingival embrasure (OGE) model, as shown in Fig. 1 . The sample size was determined using the formula E = N − T. 12 Based on this formula, the sample consists of 15 rats divided into five groups: L-PRF exudate ( *n* = 3), C-PRF ( *n* = 3), a combination of L-PRF exudate and C-PRF ( *n* = 3), HA ( *n* = 3) as treatment groups, and phosphate-buffered saline (PBS) ( *n* = 3) as the control group.

Sprague-Dawley male rats weighing between 250 and 300 g were kept in a breeding room with a 12-hour light/dark cycle at 25°C and a humidity level ranging from 64 to 80°F. During both the maintenance and treatment periods, the animals were provided with a standard pelleted diet and ad libitum access to tap water. Before treatment, the animals were anesthetized intramuscularly with ketamine hydrochloride (Ketamil; Ilium, Troylab, Australia) at a dosage of 90 mg/kg of body weight and xylazine (Xyla, Interchemie, The Netherlands) at a dosage of 10 mg/kg of body weight. 13

This study modified the OGE model described in the previous study. 13 14 Unlike the previous study, this study induces the OGE using a U-shaped 20-mm-long, 0.016-gauge stainless steel wire looped onto the mandibular incisors and secured with flowable composite resin. This wire was applied to produce lateral pressure and cause space between the mandibular incisor (OGE width). On day 7 postinduction, clinical parameters, which are OGE width and spring papilla distance (SPD), were measured using a sliding caliper. Following OGE confirmation, 20 µL of various test materials were injected 2 mm from the apex of the interdental papillae using a 30G syringe.

On day 14, the animals were euthanized, and the interdental papillae tissues between the mandibular incisors were processed. The papillae height (SPD), histological (fibroblast, blood vessels, collagen density, and epithelial thickness), and immunohistochemical (proliferating cell nuclear antigen [PCNA] expression) parameters were used to determine interdental papillae regeneration. The SPD on day 7 was analyzed statistically using a one-way analysis of variance (ANOVA). The Kruskal–Wallis' test was used to analyze the OGE width on days 7 and 14, and the SPD on day 14, which were not normally distributed. The least significant level is less than 0.05.

### Histological Study and Data Analysis

The number of fibroblasts, blood vessels, collagen density, and epithelial thickness were assessed through histological examinations. Paraffin blocks were cut into slices of 3-µm thickness and stained with Masson's Trichrome Goldner (Bio-Optica, Milano, Italy). In a double-blind study, observations were conducted by two reliable independent observers. Each preparation was evaluated in five fields of view at ×400 magnification using an Optilab camera and ImageJ software. The histological data were normally distributed and were analyzed statistically using a one-way ANOVA. Tukey's honestly significant difference (HSD) test was employed to compare the differences between the two groups. A significance level of less than 0.05 indicated a statistically significant difference.

### Immunohistochemical Study and Data Analysis

To perform immunohistochemistry, a 3-µm-thick paraffin block is cut and dried at 37°C before being heated in a heater at 60°C for 30 to 60 minutes. The tissue preparations were performed, and the PCNA primary antibody was applied based on the instructions. Two reliable, independent observers (reliability value >80%) counted the number of brown cell nuclei in a double-blind method to detect positive PCNA expression. 14 All samples were evaluated in five fields of view at ×400 magnification using an Optilab camera and Image J software. The immunohistochemical data followed a normal distribution and were analyzed statistically using a one-way ANOVA. Tukey's HSD test was used to compare the differences between the two groups. A significance level of less than 0.05 indicated a statistically significant difference.

## Results

### Cell Counts of L-PRF Layer

The standard procedure for preparing L-PRF resulted in four distinct layers above the layer of red blood cells. Test findings indicated that the C-PRF layer contains the highest concentration of platelets and leukocytes. Additionally, the combination of C-PRF and L-PRF exudate had more platelets and leukocytes than the L-PRF exudate layer alone. In contrast, the platelet-poor plasma layer lacked platelets and leukocytes ( Table 1 ).

**Table 1:** Platelet and leucocyte count of PRF layers

PRF layer	Number of cells (10 ^9^ )	
	Platelet	Leucocyte
PPP	0	0
L-PRF exudate	17	0.3
C-PRF + L-PRF exudate	32	3.9
C-PRF	44	9.1

Abbreviations: C-PRF, concentrated platelet-rich fibrin; L-PRF, leucocyte platelet-rich fibrin; PPP, platelet-poor plasma.

### Width of OGE

On day 0 following the OGE induction, the wire was positioned parallel to the gingival margin, but a gap developed shortly after the wire was activated. By day 7 postinduction, the interdental gap had widened, and the position of the wire had moved more coronally. Notably, no pus or redness was observed on the interdental papillae. Table 2 indicates that the width of the interdental space in all groups on day 7 ( *p* -value = 0.105) and day 14 ( *p* -value = 0.137) did not show any significant differences ( *p* -value > 0.05).

**Table 2:** Comparison of clinical parameters in the modified OGE model

Parameter	Group	Descriptive	*p* -Value
OGE width on day 7	L-PRF exudate	1.3 (1.3–1.38)	0.105
	C-PRF + L-PRF exudate	1.14 (1.1–1.2)	
	C-PRF	1.2 (1.14–1.3)	
	HA	1.3 (1.1–1.32)	
	PBS	1.32 (1.3–1.78)	
OGE width on day 14	L-PRF exudate	1.58 (1.4–1.62)	0.137
	C-PRF + L-PRF exudate	1.2 (1.2–1.4)	
	C-PRF	1.4 (1.2–1.7)	
	HA	1.32 (1.2–1.38)	
	PBS	1.54 (1.38–1.72)	
SPD on day 7	L-PRF exudate	1.47 ± 0.35	0.422
	C-PRF + L-PRF exudate	1.61 ± 0.45	
	C-PRF	1.71 ± 0.50	
	HA	1.34 ± 0.55	
	PBS	2.13 ± 0.68	
SPD on day 14	L-PRF exudate	2.12 (2.10–2.54)	0.255
	C-PRF + L-PRF exudate	2.36 (2.16–2.80)	
	C-PRF	2.2 (2.19–2.80)	
	HA	2.42 (2.4–2.84)	
	PBS	2.74 (2.66–2.84)	

Abbreviations: C-PRF, concentrated platelet-rich fibrin; HA, hyaluronic acid; L-PRF, leucocyte platelet-rich fibrin; OGE, open gingival embrasure; PBS, phosphate-buffered saline; SPD, spring papilla distance.

### Height of Interdental Papillae

The height of the interdental papillae was measured as the distance between the wire and the tip of the papillae (referred to as SPD). Table 2 indicates no significant differences in the height of interdental papillae across all groups on day 7 ( *p* -value = 0.422) and day 14 ( *p* -value = 0.255).

### Histological and Immunohistochemical Parameters

**Fig. 2:**
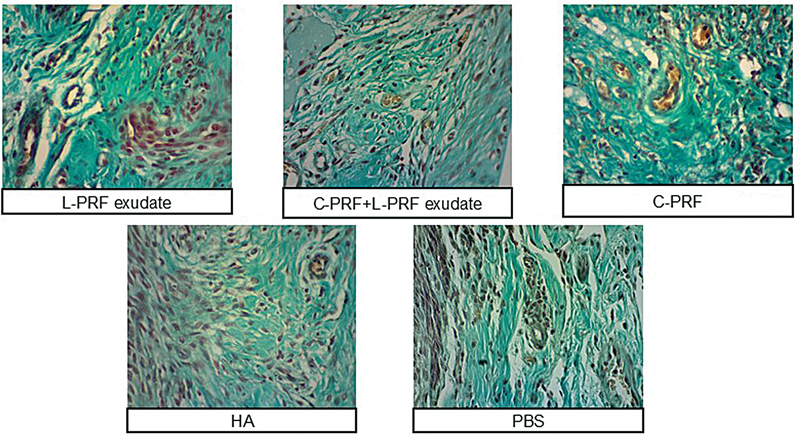
Comparison of histological parameters with Masson's Trichrome Goldner staining in studied groups. C-PRF, concentrated platelet-rich fibrin; HA, hyaluronic acid; L-PRF, leucocyte platelet-rich fibrin; PBS, phosphate-buffered saline.

**Fig. 3:**
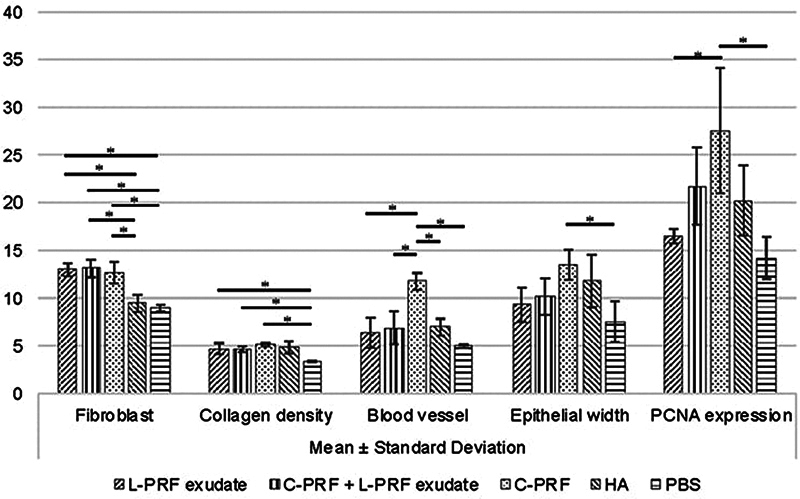
Comparison of histological and immunohistochemical parameters of interdental papillae regeneration in studied groups. The significance of a difference (*) is indicated when the p-value is less than 0.05. C-PRF, concentrated platelet-rich fibrin; HA, hyaluronic acid; L-PRF, leucocyte platelet-rich fibrin; PBS, phosphate-buffered saline.

The ANOVA test displays significant differences in various parameters: the number of fibroblast cells ( *p* -value = 0.000), the number of blood vessels ( *p* -value = 0.000), collagen density ( *p* -value = 0.003), epithelial thickness ( *p* -value = 0.045), and PCNA expression ( *p* -value = 0.017), among the test groups. The histological parameters are shown in Fig. 2 , and Tukey's HSD test in Fig. 3 . Significant differences between the two groups were observed at a *p* -value of less than 0.05.

All types of PRF demonstrate a higher number of fibroblasts compared with PBS and HA. L-PRF exudate contains significantly more fibroblasts than PBS ( *p* -value = 0.001) and HA ( *p* -value = 0.003). Similarly, C-PRF shows a higher fibroblast count than PBS ( *p* -value = 0.002) and HA ( *p* -value = 0.005). Furthermore, the combination of L-PRF exudate and C-PRF exhibits a more significant fibroblast number than PBS ( *p* -value = 0.001) and HA ( *p* -value = 0.002). However, there are no significant differences in fibroblast counts between L-PRF exudate and C-PRF ( *p* -value = 0.993), nor between the combination of both PRF types and the C-PRF group ( *p* -value = 0.971).

All types of PRF demonstrate a denser collagen structure compared with PBS. L-PRF exudate contains significantly denser collagen than PBS, with a *p* -value of 0.018. Similarly, C-PRF shows greater collagen density than PBS, with a *p* -value of 0.003. Additionally, the combination of L-PRF exudate and C-PRF exhibits denser collagen than PBS, yielding a *p* -value of 0.022. However, there are no significant differences in collagen density between L-PRF exudate and HA ( *p* -value = 0.995), C-PRF and HA ( *p* -value = 0.893), or between the combination of both types of PRF and HA ( *p* -value = 0.984).

The C-PRF group exhibited a higher number of blood vessels compared with the PBS group ( *p* -value = 0), the HA group ( *p* -value = 0.004), the L-PRF exudate group ( *p* -value = 0.002), and a combination of both types of PRF ( *p* -value = 0.004). No significant differences were observed among the other groups ( *p* -value > 0.05).

The C-PRF had a thicker epithelial width than the PBS, with a *p* -value of 0.036. However, no significant differences were observed when comparing C-PRF to the HA group ( *p* -value = 0.848), the L-PRF exudate ( *p* -value = 0.171), or a combination of both types of PRF ( *p* -value = 0.355). No significant differences were found among the other groups, as indicated by a *p* -value greater than 0.05.

This study showed that the expression of PCNA in the C-PRF group was significantly higher compared with the PBS group ( *p* -value = 0.014) and the L-PRF exudate ( *p* -value = 0.041). However, there was no significant difference between the C-PRF group and the HA group ( *p* -value = 0.235) or between the C-PRF group and the combination of both PRFs ( *p* -value = 0.427). Furthermore, the L-PRF exudate showed no significant differences from the HA group ( *p* -value = 0.775). Similarly, the combination of both PRFs also displayed no significant difference compared with the HA group ( *p* -value = 0.989).

## Discussion

Papilla reconstruction procedures can be performed nonsurgically by injecting material to enhance the gingival biotype. In this study, the injected PRF is a by-product of the L-PRF membrane preparation. Specifically, these include L-PRF exudate, C-PRF, and a combination of L-PRF exudate and C-PRF. This investigation focused on separating the layers formed during the preparation, while a previous study examined the layers formed per 1 mL of material. 11 By adjusting the separation process, this study achieved consistent results that align with previous findings.

Table 1 shows that the buffy coat layer, or C-PRF, contained the highest concentrations of platelets and leukocytes, whereas the L-PRF exudate group exhibited the lowest cell counts. In this study, we separate the PRF based on the layer produced. Unlike the previous study, which separated the layer per milliliter, 11 this procedure is advantageous because it is simple and easy to implement in clinical settings.

The regeneration process was assessed using a wire-induced OGE animal model. This model is deemed optimal, as it can replicate clinical conditions while allowing for examination of morphological and histological changes in the interdental papillae. 15 The measurements of the OGE space observed in all test groups did not differ significantly after induction (day 7) or injection (day 14). It showed that the width of the interdental papillae stayed consistent across the various test groups, allowing for effective management of individual animal variability. Controlling the individual variety of OGE space is important as this factor may influence the height of the interdental papillae.

In this study, injecting L-PRF, C-PRF exudates, or a mixture of the two forms of PRF increased interdental papillae fibroblast proliferation more than PBS. PRF is a biological material derived from a patient's blood and is rich in growth factors. PRF promotes the expression of key growth factors for gingival fibroblasts, including transforming growth factor beta (TGF-β), vascular endothelial growth factor (VEGF), bone morphogenetic protein 2, 16 and fibroblast growth factor. 17

Human L-PRF exudate fluid enhances the number of fibroblasts within 3 days of injection. 10 It contains platelet-derived growth factor (PDGF) and TGF-β, which promote fibroblast cell proliferation and migration. 18 C-PRF increases the production of PDGF and TGF-β while also stimulating proliferation and migration activities. 19 However, the study found no significant difference ( *p* > 0.05) in the number of fibroblast cells after injecting L-PRF exudate, C-PRF, and combining both PRFs. This lack of difference may be influenced by individual variations related to platelet counts and the time interval between blood collection and centrifugation. Further research is needed to explore the differences in growth factors among the various PRF layer groups and how they affect the proliferation of gingival interdental papillae fibroblasts.

The number of fibroblasts in the PRF groups was higher than that in the HA group. After the HA injection, the number of fibroblasts was similar to that observed in the PBS group. This result contradicts previous research that suggested HA injection can enhance the presence of gingival fibroblasts. 20 Several factors may have influenced this outcome, including the type of HA used, its concentration, the injection technique, and the body's cellular response. 21

The collagen density in the PRF group was higher than that in the PBS group. This suggests that the PRF group plays a significant role in forming the extracellular matrix during the regeneration of the interdental papilla. This finding aligns with previous studies indicating that PRF enhances collagen formation in periodontal tissues. 20 Additionally, PDGF and TGF-β stimulate the proliferation and migration of fibroblasts and collagen. 22

The collagen density after PRF group injection did not differ significantly ( Fig. 1 ). This finding suggests that all forms of liquid PRF used in this study have similar potential to enhance collagen density despite variations in the cell's composition of liquid PRF. Furthermore, the increased platelet count observed in C-PRF did not impact collagen density at day 7. Notably, C-PRF, derived from human blood, gradually releases its growth factor content over 10 days, with only a portion being released by day 7 postinjection. 23

It was also observed that C-PRF stimulates the production of type 1 collagen within the first 10 days of using the test material. However, the limited observation period of just 7 days in this OGE animal model poses challenges due to the physiological movement associated with the eruption of mandibular incisor teeth. 15 Therefore, developing an animal model that allows for long-term studies of collagen density is essential.

HA injection resulted in fewer fibroblasts than PRF; however, collagen density remained consistent across all forms of PRF. This implies that although their mechanisms differ, both materials can stimulate collagen fiber production equally. In the early stages of wound healing, HA does not promote fibroblast cell proliferation but influences collagen maturation and the remodeling of the extracellular matrix. 24

Several mechanisms contribute to these effects, including HA's interaction with cell surface receptors. This interaction activates signaling pathways that enhance fibroblast activity and collagen formation. 25 Additionally, HA may promote chondrocyte activity and activate mechanotransduction pathways, enabling cells to respond more effectively to mechanical stimuli within the extracellular matrix. 26

HA has been shown to stimulate collagen synthesis in the gingival area in experimental OGE models. 15 Clinically, HA injection has been demonstrated to improve the height of interdental papillae in cases of classes I and II papillae loss. 27 However, this treatment can also lead to swelling, discomfort, and a burning sensation at the injection site. 5 The therapeutic application of HA in clinical practice remains uncertain due to issues concerning predictability, long-term effects, and the stability of HA in tissues.

Rebuilding interdental papillae presents a challenge due to the narrow and delicate area and the limited number of blood vessels. 28 In this study, the number of blood vessels significantly increased following the injection of C-PRF compared with the PBS group, suggesting that C-PRF is more effective in promoting vascularization. It is crucial for supplying oxygen and nutrients during the regeneration process. 29

C-PRF boasts platelet concentrations more than 1,500% higher than standard levels. 19 Centrifugation activates platelets, which release growth factors such as PDGF, TGF-β1, endothelial growth factor, and VEGF from their α granules. 30 Additionally, C-PRF contained a higher concentration of leukocytes than both L-PRF and C-PRF exudates, further enhancing its potential benefits.

The injection of C-PRF resulted in a significantly greater number of blood vessels compared with HA. The number of blood vessels following HA injection was similar to that observed in the PBS group. It indicates that only C-PRF can enhance vascularization 7 days after injection, while HA administration does not affect vascularization.

Previous studies on the impact of HA on vascularization have produced conflicting results. Although HA can promote angiogenesis, its effectiveness is largely influenced by its molecular size and cross-linking properties. 31 High-molecular-weight HA is known for its anti-inflammatory characteristics, suppressing the body's immune response, supporting tissue healing, 32 and inhibiting angiogenesis while ensuring blood vessel stability. 33 Conversely, HA with a molecular weight of less than 200 kDa can trigger inflammation and angiogenesis by interacting with specific receptors and the receptor for hyaluronan-mediated apoptosis (RHAMM). Additionally, HA attracts stromal cells, fostering cell proliferation and migration. 34

This study found that C-PRF stimulated vascularization more effectively than other PRF groups. C-PRF contained a higher concentration of platelets and leukocytes compared with L-PRF exudates. While L-PRF exudates have platelets and leukocytes, their concentrations are lower than those in the L-PRF membrane: 6% platelets and 0.9% leukocytes. 35

Additionally, there were differences in the release of growth factors between the two types of PRF. C-PRF releases growth factors steadily starting on day 3 and continuing beyond that, 10 whereas L-PRF exudates exhibit significant growth factor release on day 3 but in smaller amount than L-PRF membranes. 36 The difference in growth factor release might influence gingival regeneration, as the growth factor induces a complex series of cellular and molecular events essential for cell proliferation. Further studies are needed to explore the variations in growth factor release among L-PRF, C-PRF, and L-PRF exudates over time.

Loss of interdental papillae can increase the risk of inflammation in periodontal tissues. The epithelium is crucial in defending periodontal tissue as a barrier against microbial invasion. 37 Unlike previous studies on wound healing, this study observed epithelialization occurring in the uninjured area of the papilla apex. Fig. 1 shows that C-PRF injection increased epithelial thickness and PCNA expression at the papilla apex compared with PBS treatment. This suggests that C-PRF may stimulate the proliferation of epithelial cells, thereby enhancing the thickness of the epithelial layer in interdental papillae.

These findings align with earlier research indicating a relationship between PCNA expression and localization in epithelial cells 38 and fibroblasts. 25 Platelet growth factors, such as EGF, have been shown to influence epithelial proliferation as active signaling molecules. 39 Additionally, the bioactive components of PRF regulate fibroblast proliferation and collagen production, fostering epithelial cell regeneration. 40

The height of the interdental papillae was not significantly different across all test groups. It suggests that the injection of the test material does not influence the clinical morphology, particularly the increase in the height of the interdental papillae observed on day 7 postinjection. 15

In the present study, the wire was positioned parallel to the apex of the papillae during the induction of OGE. However, after the induction, it appeared to have migrated more incisively. The physiological process of vertical tooth eruption can shift the observation point. Another potential contributing factor is the lateral mechanical movement observed in the OGE model. This movement promotes lateral rather than coronal regeneration as the body responds to the resulting damage or lesion.

Rat animal models have been widely used to observe short-term interdental papillae regeneration. 15 This study observed regeneration parameters on day 14, the seventh day following injection. This represents the peak of cell proliferation. However, the regeneration of interdental papillae is about immediate improvements and the sustainability of the results. Regrettably, this animal study could not be observed for an extended period due to the limitations of this animal model. This animal could not fully replicate the human condition because tight tooth contact was lost afterward when papillary augmentation was attempted, and only the short-term effects were validated due to the natural eruption of the incisors. 15 An appropriate experimental animal model is necessary to evaluate the effects of the test substance administration over an extended period.

The results of this study indicate that PRF by-products can enhance fibroblast proliferation and increase collagen density. Additionally, C-PRF offers better vascularization than HA. Despite this study's limitations, the findings are expected to provide valuable insights for optimizing the use of L-PRF in restoring interdental papillae, particularly in cases of multiple papilla loss. PRF membranes can be applied surgically in clinical settings, while the by-products generated during their preparation can also be utilized for nonsurgical purposes.

Some clinical studies showed that the injectable PRF was applied several times and observed longer to achieve the reconstruction effect. Clinical trials, especially longitudinal studies that monitor patients over extended periods, can assess the stability of interdental papillae height and esthetic outcome.

## Conclusion

This study found that the by-products of L-PRF membrane preparation—specifically, L-PRF exudate, C-PRF, and a mixture of the two—significantly increased the proliferation of gingival fibroblasts in the interdental papillae compared with HA. Among these, only C-PRF could promote vascularization in the interdental papillae while also stimulating epithelial proliferation, leading to an increase in epithelial thickness at the apex of the interdental papillae.

However, the observed cellular and molecular changes peaked on day 7 postinjection and did not result in alterations to collagen density or the height of the interdental papillae. To determine the clinical significance of these findings, a more comprehensive clinical trial with a longer observation period is needed to evaluate the potential of L-PRF as a nonsurgical therapy for reconstructing interdental papilla.
